# 937. A Clinical Prediction Tool to Determine Risk of Infection in the First Year Following Heart Transplant

**DOI:** 10.1093/ofid/ofab466.1132

**Published:** 2021-12-04

**Authors:** Whitney Perry, Jennifer Chow, Jason Nelson, David Kent, David R Snydman

**Affiliations:** 1 Division of Geographic Medicine and Infectious Diseases, Tufts Medical Center, Boston, MA; 2 Predictive Analytics and Comparative Effectiveness (PACE) Center, Institute for Clinical Research and Health Policy Studies, Tufts Medical Center, Boston, MA; 3 Tufts Medical Center, Boston, MA

## Abstract

**Background:**

Invasive infection is a dangerous complication of heart transplant (HT). No objectively-defined set of risk factors has been established which can reliably predict infection in this population. The aim of this study was to develop a clinical prediction model for use at one month post-HT with the ability to predict serious infection in the first year.

**Methods:**

A retrospective cohort study of all HT recipients at a single center between 2000 and 2018 was performed, excluding dual-organ recipients, those who died within one month of HT, and those with insufficient data. The composite endpoint included cytomegalovirus infection (CMV), herpes simplex (HSV) or varicella zoster virus infection (VZV), blood stream infection (BSI), and invasive fungal infection (IFI). The follow-up period extended from 1 month to 1 year post-HT. A least absolute shrinkage and selection operator (LASSO) regression model was fit using 13 candidate variables. A C-statistic, calibration curve, and Brier score were used to assess model performance.

**Results:**

375 patients were analyzed; 93 outcomes occurred (65 CMV, 3 HSV, 2 VZV, 28 BSI, and 15 IFI). 12 of 13 variables remained in the final model: year of transplant, age at transplant, ischemic cardiomyopathy, diabetes, immune-mediating disease, need for renal replacement therapy in first month, CMV risk status (high, intermediate, low) derived from donor-recipient serology, use of basiliximab induction, use of cytolytic agent in first month, use of ritixumab in first month, rejection treated with high-dose steroids in first month, lymphocyte count under 0.75 x10^3^cells/µL at 1 month, and inpatient status at 1 month. The C-statistic was 0.82 and Brier score 0.142. The calibration curve is shown in Figure 1.

Figure 1. Calibration plot

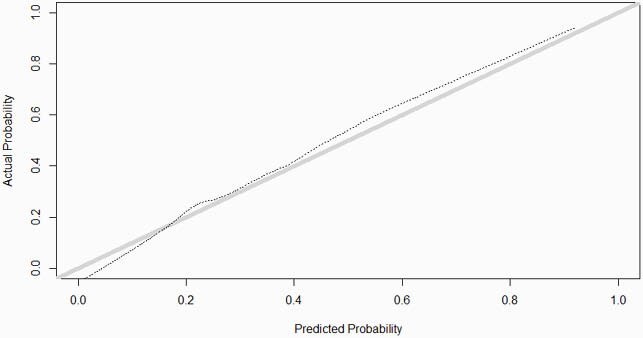

Actual versus predicted probability of infection by 1 year. Gray line = ideal Dotted line = smoothed non-parametric calibration curve

**Conclusion:**

This model synthesizes multiple highly-relevant clinical and laboratory parameters, available at 1 month post-HT, into a unified, objective, and clinically-useful prediction tool for the occurrence of serious infection in the first post-transplant year. Good discrimination and calibration are demonstrated. External validation is required before generalized use.

**Disclosures:**

**Jennifer Chow, M.D., M.S.**, **Merck** (Grant/Research Support)**Takeda** (Grant/Research Support) **David R. Snydman, MD**, **Merck** (Consultant, Advisor or Review Panel member, Research Grant or Support)**Takeda (Shire**) (Advisor or Review Panel member)

